# Functional inhibition of the RNA‐binding protein HuR sensitizes triple‐negative breast cancer to chemotherapy

**DOI:** 10.1002/1878-0261.13478

**Published:** 2023-07-19

**Authors:** Lanjing Wei, Qi Zhang, Cuncong Zhong, Lily He, Yuxia Zhang, Ahlam M. Armaly, Jeffrey Aubé, Danny R. Welch, Liang Xu, Xiaoqing Wu

**Affiliations:** ^1^ Bioengineering Program The University of Kansas Lawrence KS USA; ^2^ Department of Molecular Biosciences The University of Kansas Lawrence KS USA; ^3^ Department of Electrical Engineering and Computer Science The University of Kansas Lawrence KS USA; ^4^ Department of Pharmacology, Toxicology & Therapeutics The University of Kansas Medical Center Kansas City KS USA; ^5^ Division of Chemical Biology and Medicinal Chemistry, UNC Eshelman School of Pharmacy The University of North Carolina Chapel Hill NC USA; ^6^ Department of Cancer Biology The University of Kansas Medical Center Kansas City KS USA; ^7^ The University of Kansas Cancer Center The University of Kansas Medical Center Kansas City KS USA; ^8^ Department of Radiation Oncology The University of Kansas Medical Center Kansas City KS USA; ^9^ Higuchi Biosciences Center The University of Kansas Lawrence KS USA

**Keywords:** animal tumor model, chemoresistance, docetaxel, HuR, TNBC

## Abstract

Chemotherapy remains the standard treatment for triple‐negative breast cancer (TNBC); however, chemoresistance compromises its efficacy. The RNA‐binding protein Hu antigen R (HuR) could be a potential therapeutic target to enhance the chemotherapy efficacy. HuR is known to mainly stabilize its target mRNAs, and/or promote the translation of encoded proteins, which are implicated in multiple cancer hallmarks, including chemoresistance. In this study, a docetaxel‐resistant cell subline (231‐TR) was established from the human TNBC cell line MDA‐MB‐231. Both the parental and resistant cell lines exhibited similar sensitivity to the small molecule functional inhibitor of HuR, KH‐3. Docetaxel and KH‐3 combination therapy synergistically inhibited cell proliferation in TNBC cells and tumor growth in three animal models. KH‐3 downregulated the expression levels of HuR targets (e.g., β‐Catenin and BCL2) in a time‐ and dose‐dependent manner. Moreover, KH‐3 restored docetaxel's effects on activating Caspase‐3 and cleaving PARP in 231‐TR cells, induced apoptotic cell death, and caused S‐phase cell cycle arrest. Together, our findings suggest that HuR is a critical mediator of docetaxel resistance and provide a rationale for combining HuR inhibitors and chemotherapeutic agents to enhance chemotherapy efficacy.

## Graphical abstract

The RNA‐binding protein Hu antigen R (HuR) binds to and stabilizes its target mRNAs, promoting the translation of β‐catenin and BCL2, which enhances cell proliferation and inhibits apoptosis, respectively. The KH‐3‐induced inhibition of HuR function by disrupting HuR‐mRNA interactions accelerates mRNA decay and reduces the translation of target proteins.

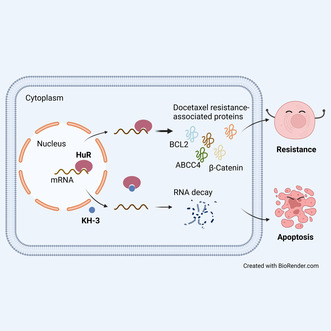

**Abbreviation**
231‐TR: docetaxel‐resistant MDA‐MB‐231 cell subline
ABCC4: ATP‐binding cassette sub‐family C member 4
BCL2: B‐cell lymphoma 2
CASPASE: cysteine‐aspartic proteases
GAPDH: glyceraldehyde‐3‐phosphate dehydrogenase
HuR: Hu antigen R
PARP: poly (ADP‐ribose) polymerases
RNP‐IP: ribonucleoprotein immunoprecipitation
RT‐qPCR: quantitative reverse transcription PCR
SD: standard deviation
SEM: standard error of the mean
TNBC: triple‐negative breast cancer
TXT: docetaxel
Wnt: wingless‐type MMTV integration site family, member 1

## 1 Introduction

Breast cancer is the most common cancer and the second leading cause of cancer death among women in the United States [1, 2]. Currently, the 5‐year relative survival rate for overall breast cancer is 90% [2]. However, the rate drops to 77% for triple‐negative [estrogen receptor negative (ER^−^), progesterone receptor negative (PR^−^), and human epidermal growth factor receptor 2 negative (HER2^−^)] breast cancer [3], which accounts for 10–15% of the total diagnosed breast cancer [4]. Compared with other subtypes of breast cancer, triple‐negative breast cancer (TNBC) has poorer prognostics, partly due to the lack of effective treatments [5, 6]. Chemotherapy is still the primary systemic treatment [7, 8] and the most effective treatment [6] for TNBC. Unfortunately, chemoresistance is a great obstacle to the successful treatment of breast cancer. Patients often develop resistance to conventional chemotherapy, and 90% of chemotherapy failures are during the invasion and metastasis of cancers related to drug resistance [9]. Therefore, to improve the survival of patients with TNBC, it is an urgent and unmet need to overcome chemoresistance and improve patients' response to current chemotherapeutics.

The Hu antigen R (HuR) or ELAVL1 (embryonic lethal, abnormal vision, Drosophila‐like protein 1) is a post‐transcriptional RNA‐binding protein and is universally expressed among all proliferating human cells [10]. HuR binds to U‐ and AU‐rich elements (AREs) mainly in the 3′‐untranslated region (UTR) and sometimes in the 5′‐UTR of target mRNAs [11, 12]. The regulatory functions of HuR on mRNAs include stabilization of target mRNAs, upregulation of the translation of target mRNAs, and inhibition of the translation of a few target mRNAs [12]. HuR promotes tumorigenesis by interacting with target mRNAs, whose encoded proteins are involved in promoting cell proliferation, improving cell survival, elevating local angiogenesis, evading immune recognition, and facilitating cancer cell invasion and metastasis [13]. Although HuR is predominantly located in the nucleus [14], it can shuttle between the nucleus and the cytoplasm, mediating different post‐transcriptional processing events [15]. The protein level of HuR, especially cytoplasmic HuR, is increased in malignancies compared to normal tissues in a broad type of cancers [16]. We previously showed that high cytoplasmic HuR was associated with high‐grade malignancy and poor clinical prognostics in breast cancer [17].

HuR overexpression is a possible mechanism for acquired chemoresistance in multiple types of cancer. The increased level of HuR is associated with chemoresistance in glioma cells [18], pancreatic cancer cells [19], and colorectal cancer cells [20]. The decrease in cytoplasmic HuR protein levels reduced the therapeutic resistance of ovarian cancer cells [21], and breast cancer cells [22]; a reduction in HuR expression resulted in chemo‐sensitized colorectal cancer cells [20], reduced tumor size [17, 18], and delayed tumor initiation *in vivo* [17]. On the contrary, chemotherapeutic agents, such as mitomycin C, oxaliplatin, cisplatin, and carboplatin, or the target therapy drugs such as ABT‐888 (PARP inhibitor), also induced the cytoplasmic accumulation of HuR [19]. It seems that there is a positive feedback loop between HuR and chemoresistance. Therefore, HuR is a potential target for enhancing chemotherapy efficacy or overcoming chemoresistance.

Docetaxel (TXT), a member of the taxane family, is currently used as a first‐line chemotherapeutic agent for TNBC. One well‐established target of docetaxel is the antiapoptotic protein BCL2. The antiapoptosis activity of BCL2 is abrogated by the phosphorylation induced by docetaxel [23, 24]. For the standard chemotherapy based on anthracycline–taxanes, 30–40% of early‐stage TNBCs develop metastasis, leading to the metastatic stage and patient death [25, 26, 27]. TNBC cells develop chemotherapy resistance through multiple mechanisms [28]. The HORMA domain‐containing protein 1 (HORMAD1) has been reported to promote docetaxel resistance in TNBC by enhancing DNA damage tolerance [29]. Hypoxia can induce docetaxel resistance through the HIF‐1α/miR‐494/Survivin signaling pathway [30]. Additionally, the kinesin family member 1 (KIF11) is critical for the proliferation and self‐renewal of docetaxel‐resistant TNBC cells [31]. To improve the antitumor efficacy of docetaxel, the combination of docetaxel with other antitumor agents has been explored in prostate cancer with all‐trans retinoic acid [24], in gastric cancer with capecitabine [32], and in TNBC with carboplatin [33], or cisplatin [34]. In addition, docetaxel was combined with target therapies in breast cancer [35], such as trastuzumab targeting HER2, bevacizumab targeting VEGF. Chemotherapy is also combined with immunotherapy, such as paclitaxel plus pembrolizumab (targeting PD‐1) to improve the survival of patients with advanced TNBC [36].

We recently reported that KH‐3, as a small molecule HuR functional inhibitor, disrupts the interactions between HuR and its target mRNAs [17]. In the current study, we evaluated the antitumor efficacy of the combination of docetaxel and KH‐3, in docetaxel‐sensitive and docetaxel‐resistant TNBC cells. The KH‐3 single‐agent treatment inhibited cell proliferation and induced apoptotic cell death. The combination of KH‐3 with docetaxel inhibited cell proliferation synergistically in several TNBC cell lines. KH‐3 also potentiated the antitumor efficacy of docetaxel in several preclinical mouse models. Mechanistically, KH‐3 treatment downregulated the expression of HuR targets b‐Catenin, and BCL2, which are found to be upregulated in docetaxel‐resistant TNBC cells. We propose that the combination of HuR inhibitors with docetaxel may be a novel therapeutic approach to enhance the chemotherapy efficacy in TNBC.

## 2 Materials and methods

### 2.1 Cell culture and reagents

The human TNBC cell line MDA‐MB‐231 (RRID: CVCL_0062) and the mouse TNBC cell line 4T1 (RRID: CVCL_0125) and EMT6 (RRID: CVCL_1923) were purchased from the American Type Culture Collection (Manassas, VA). The human TNBC cell line SUM159 (RRID: CVCL_5423) was obtained from Asterand [37]. Cells were maintained as described before [17].To obtain docetaxel (TXT) resistant cells, MDA‐MB‐231 cells were maintained in the presence of an intermittently increased concentration of docetaxel (CT‐0521, ChemieTek, Indianapolis, IN, USA). After establishing the stable resistant cells (231‐TR), cells were maintained in the presence of 2 nm docetaxel. Docetaxel was removed prior to the experimental procedure. KH‐3 was synthesized as described previously [17]. Cell lines used in this study were regularly monitored to ensure the mycoplasma‐free cells. In addition, cell lines used were regularly monitored by short‐tandem‐repeat profiling.

All the primer pairs are listed in Table S1. All the antibodies used are listed in Tables S2 and S3. Other chemical reagents used are listed in Table S4.

### 2.2 MTT‐based cytotoxicity assay

The effect of KH‐3 on cancer cell proliferation was determined by MTT‐based cytotoxicity assay as described previously. Briefly, suspended cells were seeded into the 96‐well plate with 4000 cells per well for MDA‐MB‐231 cells and 8000 cells per well for 231‐TR cells. After the 4‐day incubation in the humidified chamber at 37 °C with 5% CO2, 100 μL cell proliferation regent WST‐8 was added (Sigma Aldrich). After 2–3 h incubation at the chamber, read the absorbance at 450 nm with correction at 650 nm wavelength by the Microplate Reader (Synergy H4 Hybrid Reade, BioTek). The drug effect was assessed as the percent of inhibition compared with the untreated control. The drug concentration causing 50% inhibition was calculated using the sigmoid curve fitting by graphpad prism 8.0 (GraphPad, Inc). The combination effects of KH3 with docetaxel were determined by combination treatment MTT assay following the same protocol.

### 2.3 Clonogenic assay

Resuspended cells were seeded in the 6‐well culture plate in triplicates with 200 cells per well, and added drug to the desired concentration. The solvent, DMSO, was used as the vehicle control. The cells were maintained in the humidified chamber, and 0.5 mL FBS per 2 mL medium was added to each well on day 5. After 9–14 days of incubation, removed the medium, washed cells with PBS, and stained the cells with 0.1% crystal violet with great care to avoid disturbing the attaching cells. The total number of colonies with over 50 cells per colony was manually counted. Plating efficiency was calculated by the equation: $\text{Lating efficiency}=\frac{\text{Colony number}\;\left(\text{treat}\right)}{\text{Colony number}\;\left(\text{control}\right)}.$

### 2.4 RNA extraction and the NanoString gene expression assay

Cells or tissues after the desired treatments were collected. For the general RNA analysis, the total RNA was extracted by TRIzol Reagent (Life Technologies, cat# 15596) following the manufacturer's protocol. For the NanoString gene expression assay, the total RNA was extracted using the commercial Zymo Direct‐zol RNA MiniPrep PLUS Kit (Zymo Research, cat# R2072) following the manufacturer's protocol. The prepared total RNA was sent to NanoString Technologies (Seattle, WA, USA) for the NanoString nCounter gene expression analysis using the Tumor Signaling 360 panel. The pathway score was calculated with ncounter advanced analysis software (version 2.0.115) by the first principal component of gene expression data method following the manufacturer's user manual. The heatmap of the pathway score was plotted with Heatmapper using the Euclidean measurement method (http://www.heatmapper.ca/).

### 2.5 Quantitative reverse transcription PCR (RT‐qPCR)

The total RNA (2 μg) extracted by TRIzol Reagent was reverse transcribed into cDNA first using the commercial High Capacity cDNA Reverse Transcription Kit (Applied Biosystems, cat# 4368813). The abundance of specific mRNA was detected using the commercial PowerUP SYBR Green Master Mix Kit (Applied Biosystems, cat# A25742) in the StepOnePlus Real‐Time PCR system (Applied Biosystems). The amplified products of the primer pair were designed to be 90–200 bp, and the specificity of the fragments was verified by the melting curve analysis. The ΔΔ*C*_t_ assay was performed to calculate the relative quantification of target mRNA normalized to the housekeeping genes (*GAPDH*, *ACTB*, *RNA18SN1*, or *RNA6‐7*). All experiments were carried out in triplicates. All the primer pairs are listed in Table S1.

### 2.6 Protein extraction

For total cell lysate, cells were collected, prewashed twice with cold PBS buffer, and lysed with RIPA buffer (1% NP‐40, 0.5% Sodium deoxycholate, and 0.1% SDS in PBS) plus the protease inhibitor cocktail (1 tablet/10 mL for final working concentration, Roche, cat# 11836170001, Mannheim, Germany), and the inhibitor of serine proteases phenylmethanesulfonyl fluoride (PMSF) (1 mm final working concentration, Sigma, cat# P7626‐1G). For tumor tissue lysate, a small amount of pre‐frozen tissues was transferred into a 1.5 mL microcentrifuge tube; RIPA buffer was added to the same tube, and the tissue was ground thoroughly with a disposable pellet pestle (Thermo Fisher Scientific, USA, cat# 12‐141‐368). The lysate was kept on ice for a total of 30 min, with vortexing for a few seconds at the highest speed every 10 min. To recover the clean whole‐cell lysate, centrifuged the above reaction mixture for 20 min × 12 000 ***g*** at 4 °C, and collected the supernatant by pipetting. The commercial kit NE‐PER Nuclear and Cytoplasmic Extraction Reagents (Thermo Scientific, cat#78833) was used to extract the cytoplasmic protein lysate, following the manufacturer's protocol. The protein concentration was determined by the Bradford protein assay using the commercial Protein Assay Dye Reagent (Bio‐Rad, cat# 5000006) following the manufacturer's protocol. The prepared protein samples were stored at −80 °C, pending further examination.

### 2.7 Western blot assay

A total of 30 μg of protein was electrophoresed by the commercial SDS/PAGE gel (Genscript Biotech, cat# M00657, M00654, Piscataway, NJ, USA). Transferred the separated proteins to 0.2 μm polyvinylidene difluoride membranes (Bio‐Rad, cat# 1620177) and blocked the membrane with the Odyssey® Blocking Buffer in TBS (LI‐COR Biosciences, cat# 927‐50000) overnight at 4 °C, or with the EveryBlot Blocking Buffer for (Bio‐Rad, cat# 12010020) 30 min at room temperature. Probed the membrane with a primary antibody followed by a fluorescence‐labeled/HRP‐conjugated secondary antibody. Detected the target protein‐specific fluorescence/chemoluminescence signal with the Odyssey Fc Imaging System (LI‐COR Biosciences). All the antibodies used are listed in Tables S2 and S3.

### 2.8 Ribonucleoprotein immunoprecipitation (RNP‐IP)

To verify the interaction between HuR protein and its target mRNAs, the ribonucleoprotein immunoprecipitation (RNP‐IP) was performed following the previously described protocol with a few modifications. (a) Cells pretreated with KH‐3 for 6 h were collected and lysed with RIPA buffer containing the RNase inhibitor (100 unit·mL^−1^ for final working concentration, Invitrogen, cat# 10777‐019), plus the protease inhibitor cocktail (Roche, cat# 11836170001), and the inhibitor of serine proteases phenylmethanesulfonyl fluoride (PMSF). The whole‐cell lysate was obtained following the same protocol described in protein extraction. (b) The protein lysate was prewashed with 25 μL of Dynabeads Protein G (Invitrogen, cat# 10003D). The mixture of cell lysate and beads was incubated overnight at 4 °C with gentle rotation to exclude nonspecific bindings. (c) Collected the supernatant and determined the protein centration as described in protein extraction. Normalized the protein concentration to 1–2 mg·mL^−1^ with freshly prepared lysis buffer. Took 50–100 μL protein lysate and stored it with TRIzol Reagent, which would be used for the input control. Transferred 1 mg total protein to a new tube, added 1 μg HuR antibody, or 1 μg of IgG from the same species of HuR to the same tube, and incubated for 1 h at 4 °C with gentle rotation. (d) Transferred the supernatant in step 3 to a new tube, added new beads to the same tube (50 μL·mg^−1^ total protein), and incubated for 3 h at 4 °C with gentle rotation. (e) Washed the beads by discarding the supernatant, resuspending with 500 μL washing buffer I, and incubating for 20 min at 4 °C with gentle rotation. Repeated the washing step with washing buffers ii and iii. (f) Removed the supernatant, added 0.5–1 mL TRIzol Reagent to the tube, vortexed the mixture, and stored the sample at −80 °C, pending RNA extraction, and RT‐qPCR analysis.

### 2.9 Cell viability detection

The cell viability was determined by the AO/PI staining assay (Nexcelom Bioscience, cat# CS2‐0106‐5mL, Lawrence, MA, USA) following the manufacturer's suggested protocol. Briefly, total cells were harvested by trypsinization and centrifugation and resuspended with DMEM medium. For AO/PI staining, took 25 μL of AO/PI staining solution to a 1.5 mL Eppendorf tube; added 25 μL of the cell suspension to the same tube and mixed by pipetting up and down; immediately loaded 20 μL mixture into a disposable counting chamber (SD100 Slides, Nexcelom Biosciences, Lawrence, MA, USA) and analyzed with the cell counter (Cellometer Vision, Nexcelom Bioscience).

### 2.10 Time‐lapse live‐cell imaging

The EVOS cell imaging system (EVOS FL Auto, Thermo Fisher) was used to monitor the time‐lapse images of the morphology of cells receiving treatments. Cells were seeded in a 12‐well plate or a 6‐well plate. After overnight culture, cells were treated and transferred into the on‐stage incubation (6% CO_2_, 37 °C), maintaining for 96 h. Cell imagines were taken automatedly every 20 min, recorded, and used to generate movies.

### 2.11 Cell cycle analysis by flow cytometry

MDA‐MB‐231 cells were treated with 5 or 10 μm KH‐3. All floating and adhering cells were harvested by trypsinization, then centrifuged for 5 min × 200 ***g*** at 4 °C, and resuspended with 0.5 mL DPBS with 0.5% (v/v) fetal bovine serum. Cells were fixed in a final concentration of 75% ethanol by dropwise adding 1.5 mL pre‐cold ethanal while gently vortexing the sample. The cells were stored at −20 for more than 24 h, pending the propidium iodide (PI) DNA staining. The fixed cells were washed once with 2 mL DPBS with 0.5% (v/v) fetal bovine serum, and pelleted by centrifuging for 5 min × 400 ***g*** at 4 °C. Finally, the cells were resuspended with 0.5 mL PI staining solution (50 μg·mL^−1^ PI, 100 μg·mL^−1^ RNAse) and incubated for 30 min at room temperature in the dark. Stained cells were analyzed by the BD Accuri C6 Plus flow cytometer (BD Science). A total of 1 × 10^5^ events (FL2 > 80 000) for each sample were counted and calculated by the flowjo software using the Watson model (Version 10.8.1). Each experiment was performed in triplicate.

### 2.12 Synergism analysis

To assess the synergistic effects of the combination, the synergy score of two compounds was calculated using the synergyfinder web tool (http://www.synergyfinder.org/) with the HSA method. The combination MTT‐based assay with a fixed mole ratio of KH‐3 to docetaxel was performed, and the combination index (CI) was calculated by Compusyn [38].

### 2.13 Animal tumor models

Four‐ to six‐week‐old female athymic NCr‐nu/nu mice or 6–8‐week‐old female BALB/c mice purchased from Charles River Laboratories (Wilmington, MA, USA) were used to assess the *in vivo* combination effects of KH‐3 and docetaxel. Suspended MDA‐MB‐231 cells (0.85 × 10^6^ cells), 231‐TR cells (1.0 × 10^6^ cells), or mouse TNBC EMT6 cells (0.15 × 10^6^ cells) in exponential‐stage growth were injected into the third mammary fat pad of each mouse. When the size of MDA‐MB‐231 xenografts reached around 30–50 mm^3^, mice were randomized into four groups with 10 tumors/group. Each group received one of the following treatments intraperitoneally (i.p.) or intravenously (i.v.): (a) KH‐3, i.p., 50 mg·kg^−1^, QD5 × 4 weeks; (b) KH‐3, i.p., 50 mg·kg^−1^, QD5 × 4 weeks, plus docetaxel, i.v., 10 mg·kg^−1^ (1st week), 7.5 mg·kg^−1^ (2nd week), 5 mg·kg^−1^ (3rd week), QW × 3 weeks; (c) docetaxel, i.v., 10 mg·kg^−1^ (1st week), 7.5 mg·kg^−1^ (2nd week), 5 mg·kg^−1^ (3rd week), QW × 3 weeks; (d) no treatment. For 231‐TR xenografts, the same grouping and the treatment schedule were performed with an increased‐dose schedule of docetaxel (10 mg·kg^−1^, QW).

For EMT6 allografts, a similar grouping and a modified treatment schedule were performed, (a) KH‐3, i.p., 40 mg·kg^−1^, QD5 × 4 weeks; (b) KH‐3, i.p., 40 mg·kg^−1^, QD5 × 4 weeks, plus docetaxel, i.v., 10 mg·kg^−1^ (1st week), 7.5 mg·kg^−1^ (2nd week), QW × 2 weeks; (c) docetaxel, i.v., 10 mg·kg^−1^ (1st week), 7.5 mg·kg^−1^ (2nd week), QW × 2 weeks; and (d) no treatment. From the first day of treatment, the tumor size and body weight was monitored twice a week. The tumor volume was calculated based on the equation: $\text{Tumor volume}=\frac{\text{Length}\times {\text{Width}}^{2}}{2}$. All the animal experiments performed in this research were approved by the Institutional Committee for the Use and Care of Animals of the University of Kansas (Authorization # AUS205‐2). Mice were maintained under a specific pathogen‐free animal facility with a 12 h on/12 h off light cycle. Cages and beddings were autoclaved before use. Mice were provided with free access to standard diet and sterile water. All experiments were started at least 3 days after the animals arrived at the animal room facility.

### 2.14 Statistical analysis

One‐way or two‐way ANOVA test was used to determine the significant differences by prism software 8.0 (GraphPad). For animal experiments, Kaplan–Meier analysis followed by a log‐rank test was used to assess the tumor growth suppression by Prism software 8.0. The applied statistical analysis for each data set was indicated in the figure legends. *P* < 0.05 is considered to be statistically significant. All *in vitro* experiments, unless otherwise specified, were repeated at least three times.

## 3 Results

### 3.1 HuR functional inhibitor KH‐3 inhibited TNBC cells proliferation and reduced cell viability

**Fig. 1:**
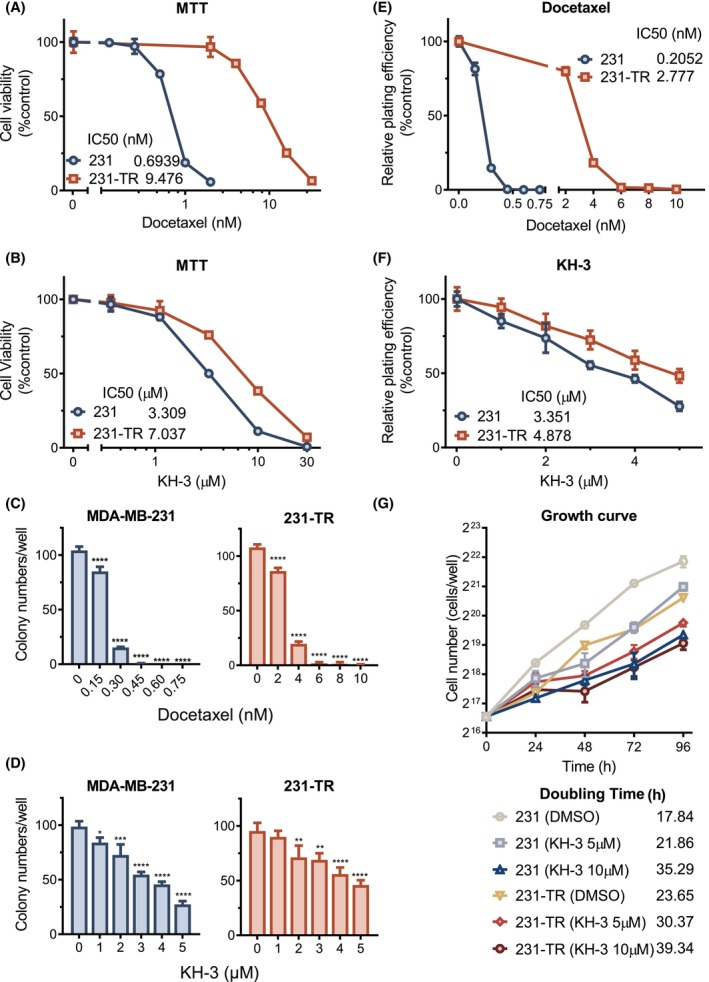
HuR functional inhibitor KH‐3 inhibited TNBC cell proliferation and reduced cell viability. (A) The cell viability curves determined by the MTT‐based cytotoxicity assay of MDA‐MB‐231 and 231‐TR cells treated with docetaxel or (B) KH‐3. (C, D) The colony number determined by the clonogenic assay of MDA‐MB‐231 and 231‐TR cells treated with (C) docetaxel or (D) KH‐3. Ordinary one‐way ANOVA test, *P < 0.05, **P < 0.01, ***P < 0.001, ****P < 0.0001 (*n* = 3). (E) The relative plating efficiency of cells treated by docetaxel in panel C. (F) The relative plating efficiency of cells treated by KH‐3 in panel D. Plating efficiency was calculated by the equation: $\text{Plating efficiency}=\frac{\text{Colony number}\;\left(\text{treat}\right)}{\text{Colony number}\;\left(\text{control}\right)}.$ (G) The growth curve of viable cells determined by trypan blue solution staining assay. Cells were treated with DMSO (vehicle control) or KH‐3 (5 or 10 μm) at time zero. All results are shown as mean ± SD of three replicates, unless otherwise specified.

The cytotoxicity of KH‐3 in TNBC cells was investigated using the cell line MDA‐MB‐231 (231) and its derived docetaxel (TXT) resistant cell‐subline (231‐TR). The MTT‐based cytotoxicity assay was performed to compare the sensitivity of MDA‐MB‐231 and 231‐TR to docetaxel or KH‐3 (Fig. 1A,B). The IC50 of docetaxel for 231‐TR cells was 9.48 nm, which was over 10‐fold higher than the IC50 for MDA‐MB‐231 (0.69 nm) (Fig. 1A), indicating the docetaxel resistance of 231‐TR cells. In contrast, 231‐TR showed a slightly higher IC50 than KH‐3: it showed an IC50 of 7.04 μm compared with 3.31 μm of the parental cell line (Fig. 1B).

To evaluate the effects of KH‐3 on cell proliferation, the clonogenic assay was performed. The colony number decreased with the increased concentration of docetaxel, or KH3 alone (Fig. 1C,D and Fig. S1A,B). The IC50 of docetaxel in inhibiting the plating efficiency of 231‐TR cells was 2.78 nm compared with 0.21 nm of parental cells (Fig. 1E). The IC50 of KH‐3 in inhibiting the plating efficiency was 3.35 and 4.89 μm for MDA‐MB‐231 and 231‐TR cells respectively (Fig. 1F). To further validate the proliferation inhibition effects of KH‐3, the growth curve was calculated. The doubling times for MDA‐MB‐231 and 231‐TR cells were 17.84 and 23.65 h respectively, which were extended by KH‐3 treatment in a dose‐dependent manner (Fig. 1G).

### 3.2 KH‐3 showed synergy with docetaxel *in vitro*

**Fig. 2:**
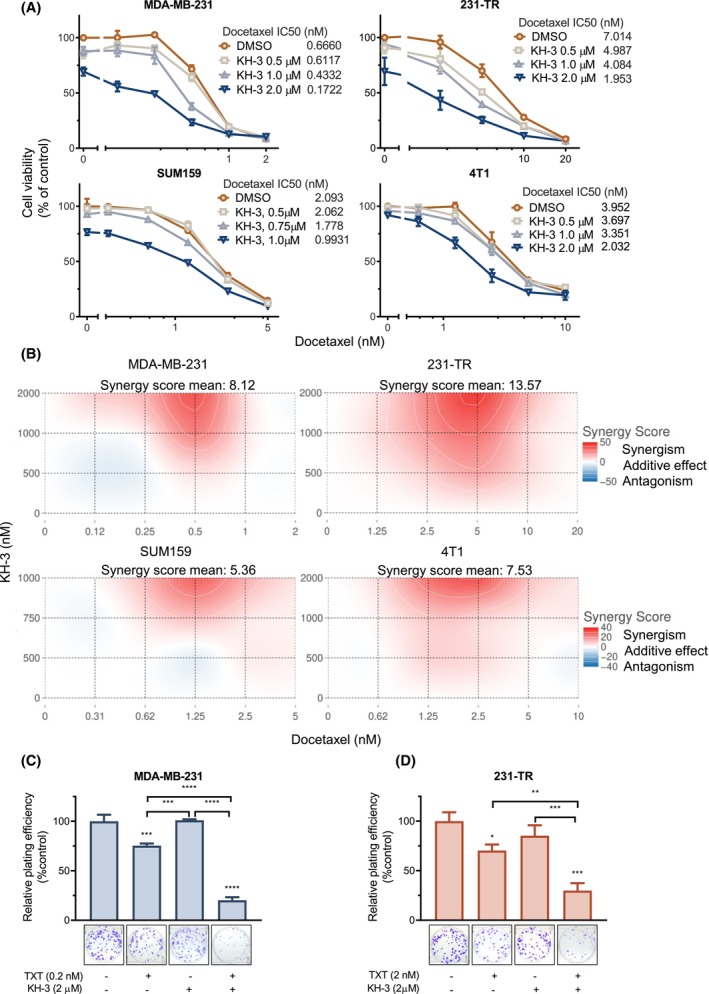
KH‐3 showed synergy with docetaxel *in vitro*. (A) The cell viability curves determined by the MTT‐based cytotoxicity assay of MDA‐MB‐231 cells, 231‐TR cells, SUM159 cells, and 4T1 cells treated with docetaxel plus KH‐3. (B) Synergy score plots of the combination of KH‐3 plus docetaxel in panel A. (C) The relative plating efficiency of MDA‐MB‐231 cells and (D) 231‐TR cells receiving KH‐3/docetaxel single‐agent treatment, or the combination treatment. Representative colony imagines were shown in panels C, D. Ordinary one‐way ANOVA test, *P < 0.05, **P < 0.01, ***P < 0.001, ****P < 0.0001. All results are shown as mean ± SD of three replicates, unless otherwise specified.

To investigate whether KH‐3 sensitized TNBC cells to the chemotherapy drug, the cytotoxic effect of the combination treatment of KH‐3 and docetaxel was determined by MTT assay in multiple TNBC cells (Fig. 2A). The cell viability curves all shifted to the left when the concentration of KH‐3 was increased, suggesting that KH‐3 enhanced the efficacy of docetaxel. And the combination treatment displayed synergistic effects (Fig. 2B). On the one hand, 231‐TR cells showed a higher synergy score than the parental MDA‐MB‐231 cells (Fig. 2B), indicating that HuR may be critical for driving cell proliferation and cell survival, especially for chemoresistant cells. In addition, the fixed molar ratio (KH‐3 to docetaxel) combination MTT assay (Fig. S2A,B) was performed to estimate the combination effects using the combination index (CI). The results validated that the combination of two drugs was synergistic in MDA‐MB‐231 cells and 231‐TR cells (Fig. S2C). More interestingly, SUM159 and 4T1 cells showed intrinsic docetaxel resistance compared with MDA‐MB‐231 (Fig. 2A); the combination treatment was synergistic in both cell lines (Fig. 2B), supporting that combination treatment is a promising approach to overcome docetaxel resistance.

A clonogenic assay was performed to assess the combined effects of KH‐3 and docetaxel on inhibiting cell proliferation (Fig. 2C,D and Fig. S3A,B). For the 231‐TR cells, docetaxel was increased by 10‐fold to achieve a similar inhibition level on relative plating efficiency compared with MDA‐MB‐231 cells. A sublethal dose of KH‐3 showed no significant effects on the plating efficiency. However, when together with docetaxel, KH‐3 significantly suppressed the plating efficiency of both cell lines more than any single‐agent treatment (Fig. 2C,D). Taken together, these data demonstrate the synergistic effects of KH‐3 and docetaxel combination on inhibiting cell proliferation in TNBC cells.

### 3.3 231‐TR cells showed the upregulated expression levels of HuR targets involved in cell proliferation and survival

**Fig. 3:**
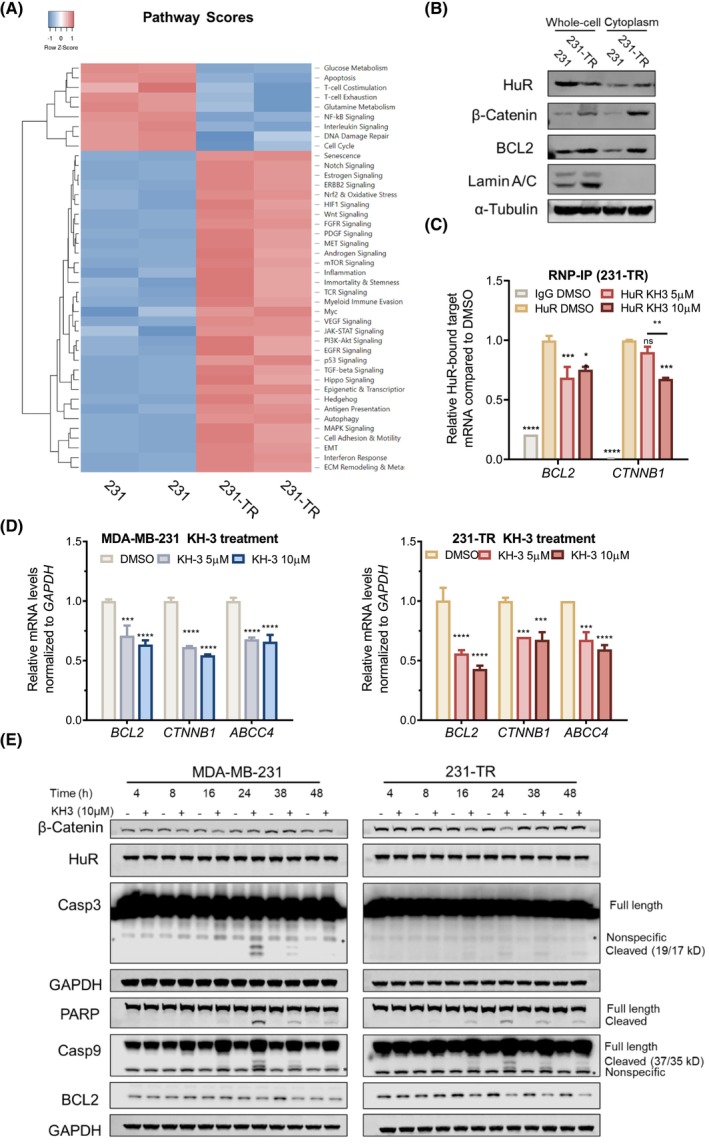
KH‐3 attenuated the expression of β‐Catenin and BCL2. (A) Heatmap of pathway scores of MDA‐MB‐231 cells and 231‐TR cells. (B) Western blot analysis of whole‐cell lysates or cytoplasmic lysates from MDA‐MB‐231 cells and 231‐TR cells. The α‐Tubulin was used as the loading control. (C) RNP‐IP analysis of HuR bound mRNAs interacted by KH‐3 in 231‐TR cells. Cell lysates of 231‐TR pretreated by KH‐3 or vehicle control (DMSO) for 6 h were subjected to RNP‐IP followed by RT‐qPCR analysis to measure the abundance of mRNAs. The enrichment of mRNAs in control IgG and HuR IP with KH‐3 treatment was compared with HuR IP with the vehicle control treatment. Two‐way ANOVA test, *P < 0.05, **P < 0.01, ***P < 0.001, ****P < 0.0001 (*n* = 2). (D) Quantitative RT‐PCR analysis on the mRNA levels of *BCL2*, *CTNNB1*, and *ABCC4* in MDA‐MB‐231 cells and 231‐TR cells treated by KH‐3 for 6 h. Two‐way ANOVA test, ***P < 0.001, ****P < 0.0001 (*n* = 2). (E) Western blot analysis of whole‐cell lysates from MDA‐MB‐231 cells or 231‐TR cells treated with KH‐3 (10 μm) for the indicated times. GAPDH was used as the loading control. All results are shown as mean ± SD. All results were performed for three repeats, unless otherwise specified.

The above data indicate that KH‐3 chemo‐sensitizes TNBC cells to docetaxel. However, the molecular mechanism of action (MOA) behind this sensitization was unknown. We conducted a gene profiling analysis using the Tumor Signaling 360 panel of the NanoString nCounter gene expression platform. The heatmap of pathway scores revealed changes in pathway regulation between parental MDA‐MB‐231 cells and 231‐TR cells (Fig. 3A). Interestingly, the apoptosis pathway was downregulated and the Wnt signaling pathway was upregulated in 231‐TR cells compared with parental MDA‐MB‐231 cells (Fig. S4A). The genes *BCL2* and *CTNNB1* are key regulators of the apoptosis and the Wnt Signaling pathway, respectively. The mRNA transcripts of both genes are direct HuR targets [18, 39], which was also confirmed by the immunoprecipitation (RIP)‐sequencing in our previous study [17]. The mRNA of *ABCC4*, encoding the multidrug resistance‐associated protein 4 (MRP4), was also a direct HuR target based on the RIP‐sequencing [17] and RNP‐IP (Fig. S4E).

The mRNA levels of *BCL2*, *CTNNB1*, and *ABCC4* were increased in 231‐TR cells compared with the parental cells (Fig. S4B). Compared with MDA‐MB‐231 cells, 231‐TR cells had increased cytoplasmic protein levels of HuR and BCL2, and β‐Catenin (the protein product of *CTNNB1*), but the whole‐cell protein level of HuR remained similar (Fig. 3B). This suggests that the increased HuR cytoplasmic accumulation, subsequently the enhanced levels of target proteins, may be critical for docetaxel resistance. Additionally, docetaxel induced HuR cytoplasmic translocation in a time‐dependent manner (Fig. S4C). The results of RNP‐IP indicated that *BCL2* and *CTNNB1* remained to be HuR targets in 231‐TR cells (Fig. 3C). Notably, KH‐3 could interrupt the interactions between HuR and mRNAs of *BCL2*, or *CTNNB1* (Fig. 3C). Therefore, a possible mechanism of action of KH‐3 in chemo‐sensitization is to regulate BCL2 and β‐Catenin.

### 3.4 KH‐3 attenuated the expression levels of β‐Catenin and BCL2

To further investigate the regulation of KH‐3 on the expression of HuR‐downstream targets BCL2 and β‐Catenin, both mRNA and protein levels were examined. KH‐3 significantly downregulated the mRNA levels of *BCL2*, *CTNNB1*, and *ABCC4* in MDA‐MB‐231 cells and 231‐TR cells (Fig. 3D). A similar trend of downregulation was detected using different housekeeping genes (*ACTB*, *RNA18SN1*, and *RNU6‐7*) for normalization (Fig. S4F). Western blot analysis was performed to measure the protein level changes of BCL2, β‐Catenin, and proteins involved in downstream events of the BCL2‐regulated caspase activation. The time‐course analysis showed that the protein level of β‐Catenin was decreased after 16 h of KH‐3 treatment and recovered later in both cell lines (Fig. 3E). Loss of BCL2 was detected after 24 h of KH‐3 treatment in 231 cells, and 16 h of KH‐3 treatment in 231‐TR cells (Fig. 3E). The downstream proteins of the BCL2‐regulated caspase activation, including Caspase‐9, Caspase‐3, and PARP, were cleaved in a time‐dependent manner (Fig. 3E). The cleavage peaked at 24 h for time points tested in both cell lines. In addition, KH‐3 did not alter the protein level of HuR. These results demonstrate that KH‐3 downregulates BCL2 and β‐Catenin and induces the caspase activation/PARP inactivation in a time‐dependent manner, subsequently leading to apoptotic cell death.

To test the potency and specificity of KH‐3 in inhibiting BCL2 and β‐Catenin, cells were treated for 24 h with a range of doses of KH‐3 (Fig. S4D). The lowest dose, 2.5 μm, did not reduce the protein levels of β‐Catenin and BCL2 or induce the visible cleavage of Caspase‐9, Caspase‐3, and PARP in both cell lines. When the dose was increased to 5 μm, a slight decrease in β‐Catenin and trace cleavage of Caspase‐9, Caspase‐3, and PARP were detected. For both cell lines, the loss of β‐Catenin was evident by 10 μm KH‐3 treatment; the noticeable cleavage of Caspase‐9, Caspase‐3, and PARP was detected. The protein levels of BCL2 were readily downregulated by 10 μm KH‐3 treatment in 231‐TR cells. Together, the data indicate the dose‐dependent activity of KH‐3 in downregulating β‐Catenin and BCL2.

### 3.5 KH‐3 induced apoptosis in TNBC cells

**Fig. 4:**
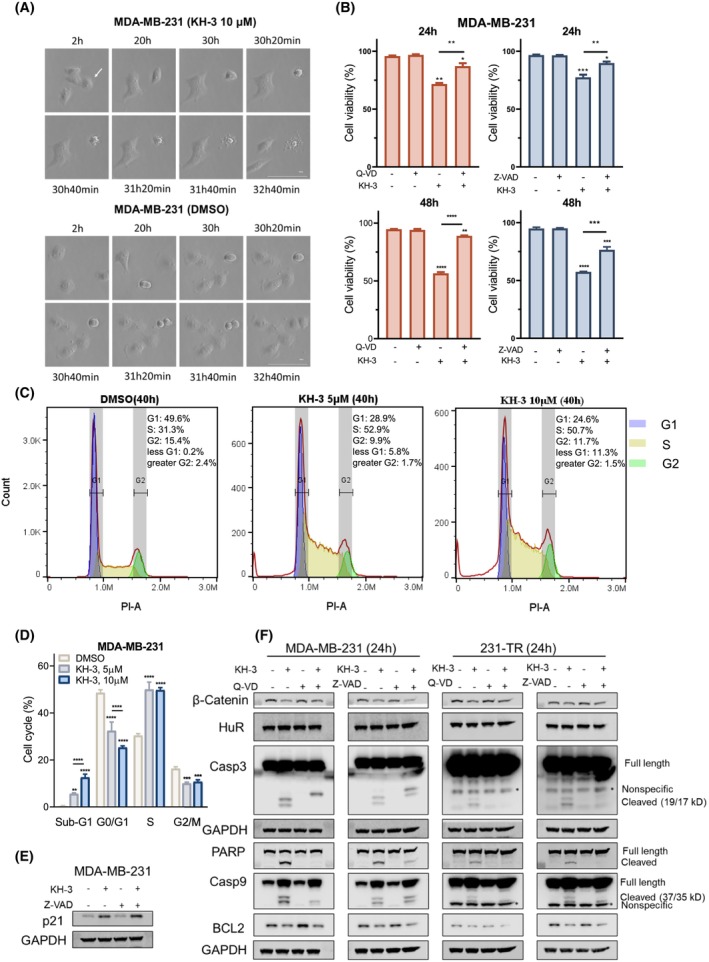
KH‐3 induced apoptosis in TNBC cells. (A) Representative time‐lapse images of the morphology of MDA‐MB‐231 cells treated by KH‐3 (10 μm) or DMSO (vehicle control). The white arrow indicates one representative cell undergoing apoptotic death. 40× microscope magnification. Scale bar 100 μm. (B) The viability of MDA‐MB‐231 cells treated by KH‐3 (10 μm) for 24 or 48 h. Cells were pretreated with Z‐VAD‐FMK (40 μm) or Q‐VD‐Oph (40 μm) for 1 h before the KH‐3 treatment. The cell viability was determined by AO/PI staining assay. Ordinary one‐way ANOVA test, *P < 0.05, **P < 0.01, ***P < 0.001, ****P < 0.0001 (*n* = 2). (C) The histograms of cell cycle analysis in MDA‐MB‐231 cells. Cells treated with DMSO (vehicle control), KH‐3 (5 μm), or KH‐3 (10 μm) for a total of 40 h were harvested, stained with PI, and analyzed by flow cytometry. The experiment was performed for three replicates, and similar results were observed. The representative results were shown. (D) Quantification of the cell cycle analysis for the treatment groups in panel C. Two‐way ANOVA test, **P < 0.01, ***P < 0.001, ****P < 0.0001. (E, F) Western blot analysis of whole‐cell lysates of cells treated by KH‐3 (10 μm) for 24 h with 1 h pretreatment of Z‐VAD‐FMK (40 μm) (E) or with 1 h pretreatment of Z‐VAD‐FMK (40 μm) or Q‐VD‐Oph (40 μm) (F). GAPDH was used as the loading control. Results were performed for three repeats, and the representative results were shown. All results are shown as mean ± SD of three replicates, unless otherwise specified.

Real‐time monitoring of the morphology of MDA‐MB‐231 cells receiving KH‐3 treatment showed that cells stopped generating daughter cells (Fig. 4A), reconfirming the proliferation suppression activity of KH‐3. On the contrary, the lysed cell, as indicated by the arrow, displayed a typical apoptotic cell death: cell shrinkage, and membrane blebbing (Fig. 4A). To confirm that KH‐3 could induce apoptosis, two caspase inhibitors were used to inhibit the activity of caspases. The Caspase‐1‐, 3‐, 8‐, and 9‐specific inhibitor Q‐VD‐Oph and the pan‐caspase inhibitor Z‐VAD‐FMK significantly attenuated the KH‐3‐induced cell death (Fig. 4B).

To further validate the apoptotic cell death, the genomic DNA fragments (sub‐G1) generated by endonucleolytic cleavage during apoptosis were examined. The cell cycle analysis revealed that the percentage of sub‐G1 was 0.2%, 5.8%, and 11.3% in the DMSO, KH‐3 (5 μm), and KH‐3 (10 μm) treated samples, respectively (Fig. 4C and Fig. S5). KH‐3 treatment resulted in a definitive accumulation in the sub‐G1 phase versus control treatment (Fig. 4D). On the contrary, the percentage of cells in the S phase was 31.3%, 52.9%, and 50.7% in the DMSO control, KH‐3 (5 μm), and KH‐3 (10 μm) treated groups, respectively (Fig. 4C). Therefore, the KH‐3 treatment resulted in a significant accumulation of cells in the S phase. Subsequently, the protein level of the cyclin‐dependent kinase inhibitor p21 was evidently increased by 24 h treatment of KH‐3 in MDA‐MB‐231 cells (Fig. 4E). The induction or overexpression of p21 is related to S‐phase arrest [40, 41]. These findings indicate that KH‐3 induces the sub‐G1 phase accumulation and causes the S‐phase arrest.

To examine the action of caspase inhibitors in rescuing cells from death, we analyzed the protein level changes caused by KH‐3 with or without caspase inhibitors. For both cell lines (Fig. 4F), Q‐VD‐Oph attenuated the cleavage of Caspase‐9 and abrogated the detectable cleavage of Caspase‐3 and PARP; in addition, Z‐VAD‐FMK treatment abrogated the detectable cleavage of Caspase‐3 and attenuated the cleavage of PARP but showed no effects on inhibiting the cleavage of Caspase‐9. Taken together, the caspase inhibition rescued cells from KH‐3‐induced cell death, identifying the caspase activation as a major mechanism of the chemo‐sensitization activity of KH‐3.

### 3.6 KH‐3 restored the TNBC response to docetaxel in 231‐TR cells by inhibiting cell proliferation and facilitating docetaxel‐induced apoptosis

**Fig. 5:**
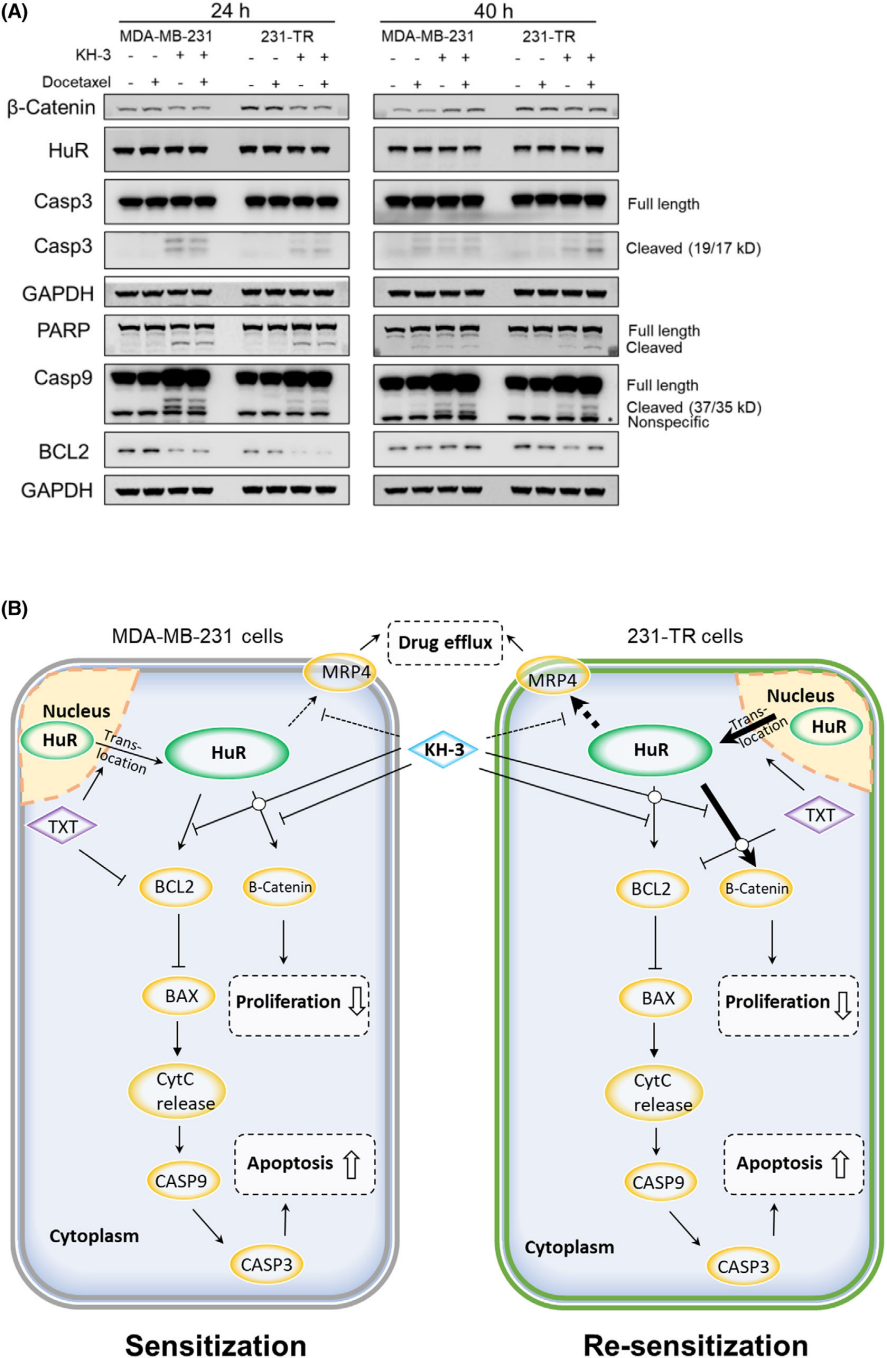
KH‐3 restored the efficacy of docetaxel in 231‐TR cells by inhibiting cell proliferation and facilitating docetaxel‐induced apoptosis. (A) Western blot analysis of whole‐cell lysates of cells treated by DMSO (vehicle control), docetaxel (5 nm), KH‐3 (10 μm), or the combination of docetaxel and KH‐3 for 24 or 40 h. GAPDH was used as the loading control. The experiment was repeated three times and representative western blot results from one experiment were shown. (B) Proposed working model of chemotherapy sensitization by the HuR functional inhibitor KH‐3.

We sought to further reveal the molecular mechanisms of action behind the chemo‐sensitization activity of KH‐3. For the KH‐3 alone or combined treatment with docetaxel for 24 h, the attenuated protein level of β‐Catenin was detected in MDA‐MB‐231 cells compared with the vehicle control (Fig. 5A, left); on the contrary, the increased protein level of β‐Catenin in 231‐TR cells was depleted to the ‘baseline level’ of parental MDA‐MB‐231 cells treated by DMSO (Fig. 5A, left). Altogether, KH‐3 synergizes docetaxel antitumor activity by suppressing the protein level of β‐Catenin at the early stage of treatment.

BCL2 is an antiapoptotic protein and tightly regulates the intrinsic apoptosis pathway. Docetaxel did not alter the overall protein level of BCL2 in both cell lines (Fig. 5A). But for the KH‐3 alone or combined with docetaxel treatment for 24 h, the loss of BCL2 was evident in MDA‐MB‐231 cells, and near complete depletion was detected in 231‐TR cells (Fig. 5A, left). KH‐3 continued depleting the protein level of BCL2 in 231‐TR cells receiving KH‐3 treatment alone for 40 h (Fig. 5A, right). It appeared that 231‐TR cells responded to KH‐3 better than MDA‐MB‐231 cells. The better response of 231‐TR cells to KH‐3 reemphasizes the critical roles of HuR in mediating chemoresistance.

We also analyzed proteins engaging in the downstream events of BCL2‐regulated caspase activation. For 24 h treatment, no detectable cleavage of Caspase‐9, Caspase‐3, and PARP was detected in docetaxel sing‐agent treatment, but the cleavage was detected in KH‐3 alone and the combination treatment (Fig. 5A, left). For 40 h treatment in MDA‐MB‐231 cells, docetaxel single‐agent treatment induced the cleavage of Caspase‐9, Caspase‐3, and PARP (Fig. 5A, right). For 40 h treatment in 231‐TR cells, the cleavage of Caspase‐9, Caspase‐3, and PARP was undetectable in docetaxel treatment alone; but the cleavage was induced by KH‐3 treatment alone and further enhanced by the combination treatment (Fig. 5A, right), suggesting that KH‐3 may overcome the HuR‐mediated resistance to chemotherapy‐induced apoptosis. The working model of chemotherapy sensitization by the HuR functional inhibitor KH‐3 is shown in Fig. 5B. These findings support that KH‐3‐mediated downregulating BCL2 may be a key event engaging in overcoming docetaxel resistance.

### 3.7 The combination of KH‐3 and docetaxel showed synergy in delaying tumor growth and improving animal survival

**Fig. 6:**
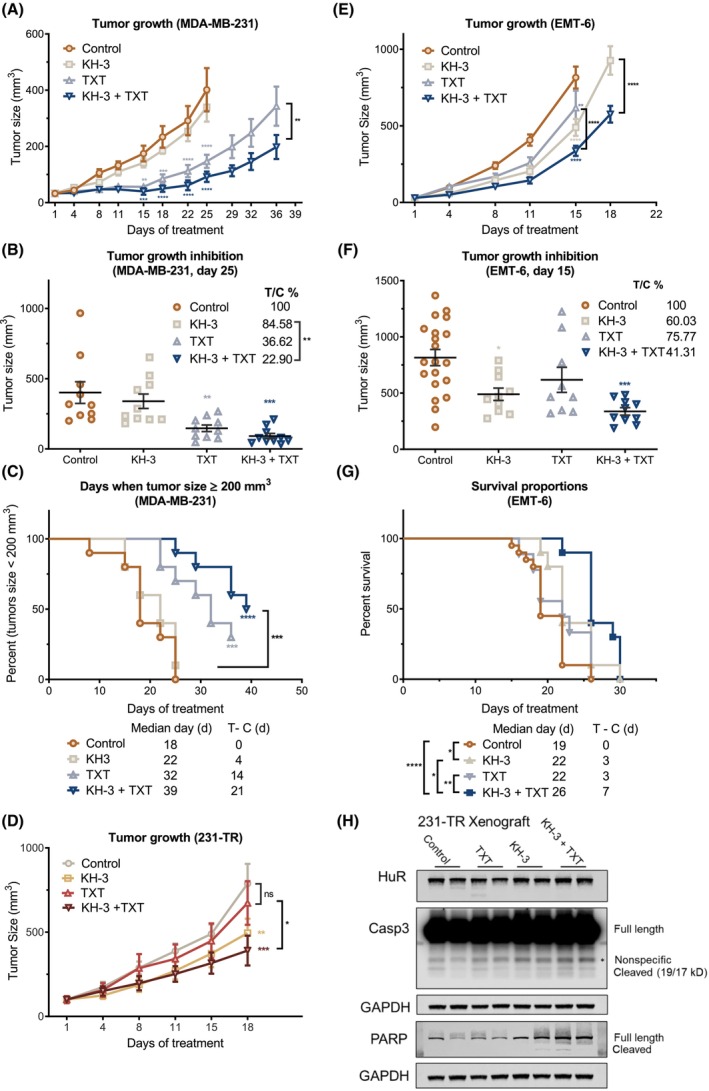
Combination of KH‐3 and docetaxel showed synergy in suppressing tumor growth *in vivo*. (A) Growth curves of tumors in the MDA‐MB‐231 xenograft model with indicated treatments. Two‐way ANOVA test, **P < 0.01, ***P < 0.001, ****P < 0.0001, *n* = 10. (B) The size of individual tumors from panel A at 25 days post‐treatment. Tumor growth inhibition (T/C %) defined as the ratio of the mean tumor size of the treated to the control group, was calculated, and listed in the figure. One‐way ANOVA test, **P < 0.01, ***P < 0.001, *n* = 10. (C) Kaplan–Meier analysis of days required when individual tumor size reached 200 mm^3^ from panel A. Log‐rank test, ***P < 0.001, ****P < 0.0001, *n* = 10. (D) Growth curves of tumors in the 231‐TR xenograft model with indicated treatments. Two‐way ANOVA test, *P < 0.05, **P < 0.01, ***P < 0.001. *n* = 8. (E) Growth curves of tumors in the EMT‐6 allograft model with indicated treatments. Two‐way ANOVA test, **P < 0.01, ****P < 0.0001, *n* = 20 for the control, *n* = 10 for the KH‐3 and the KH‐3 + TXT, and *n* = 9 for the TXT treated group. (F) The size of individual tumors from panel E at 15 days post‐treatment. One‐way ANOVA test, *P < 0.05, ***P < 0.001. (G) Kaplan–Meier analysis of survival days of mice from panel E. Log‐rank test, *P < 0.05, **P < 0.01, ****P < 0.0001. (H) Western blot analysis of tissue lysates of the 231‐TR xenograft from panel D. GAPDH was used as the loading control. All other results are shown as mean ± SEM, unless otherwise specified.

To determine the *in vivo* combination efficacy of KH‐3 and docetaxel, the mammary fat pad xenograft models of MDA‐MB‐231 and 231‐TR were utilized. The tumor growth curves of MDA‐MB‐231 xenografts showed that the combination therapy significantly suppressed the tumor growth compared with docetaxel single‐agent therapy (Fig. 6A). Moreover, at 25 days post‐therapy, when the mice from the control group reached the endpoint of euthanasia, the combination of KH‐3 and docetaxel showed a significantly enhanced tumor growth inhibition compared with the control, and KH‐3 single‐agent therapy (Fig. 6B). According to the Kaplan–Meier analysis of the time required for individual tumors to reach 200 mm^3^ (Fig. 6C), the median day for KH‐3, docetaxel, and KH‐3 plus docetaxel therapy was delayed by 4, 14, and 21 days, respectively. The body weight of mice from all groups tended to increase (Fig. S6A). Docetaxel alone significantly inhibited the growth of MDA‐MB‐231 xenografts at 15 days post‐therapy (Fig. 6A), and a higher dose of docetaxel showed no significant growth suppression on 231‐TR xenografts (Fig. 6D). However, tumor growth was significantly inhibited by KH‐3 alone, which was further inhibited by the combination therapy (Fig. 6D).

We expanded the efficacy study using the mouse syngeneic tumor model of EMT6, which is resistant to docetaxel treatment, to mimic the clinical intrinsic resistance. The combination therapy significantly suppressed the tumor growth compared with any single‐agent therapy at the end of the treatment (Fig. 6E). Although docetaxel alone showed no significant effects on inhibiting tumor growth, the KH‐3 alone and the combination treatment significantly inhibited the tumor growth (Fig. 6F). In addition, the KH‐3 or docetaxel alone delayed the median survival day of mice from 19 to 22 days, and the combined treatment further delayed the death to 26 days (Fig. 6G).

The results of the western blot analysis on the total protein lysates from 231‐TR xenografts showed that docetaxel alone did not induce the cleavage of PARP. However, KH‐3 alone or KH‐3 plus docetaxel induced the cleavage of it (Fig. 6H). In addition, KH‐3 induced the cleavage of Caspase‐9 in MDA‐MB‐231 xenografts (Fig. S6C). These data further support the role of HuR functional inhibitors, such as KH‐3, as a docetaxel sensitizer, especially in the docetaxel‐resistant TNBC cells.

## 4 Discussion

The primary systemic treatment for TNBC remains chemotherapy, but chemoresistance greatly impairs the efficacy of this approach. In order to improve the survival and prognosis of TNBC patients, there is an urgent clinical need to increase the effectiveness of chemotherapy and search for new therapeutic agents. In recent decades, advances have been achieved in the systemic target therapy for TNBC such as PARP inhibitors, antiandrogen agents, antibody‐drug conjugates, and immune‐checkpoint inhibitors [7, 42], providing new options when patients no longer responded well to chemotherapy drugs. However, the resistance to the PARP inhibitor, the lack of the androgen receptor (45–88% in TNBC) [42], or the rare expression of PD‐L1 in noninflamed tumor cells [43] all dampen the therapeutic efficacy and limit the application of those approaches in TNBC. The standard of care treatment of TNBC is still chemotherapy [7], thus enhancing the efficacy of chemotherapy is the focus of the current study. We exploited mechanisms of acquired docetaxel resistance in TNBC cells and revealed that the HuR function inhibitor (KH‐3) would interrupt these mechanisms. The combination treatment of KH‐3 and docetaxel inhibited the proliferation of TNBC cells and facilitated docetaxel‐induced apoptosis.

Cell proliferation was attenuated by KH‐3 single‐agent treatment. The Wnt‐β‐Catenin signaling pathway regulates cell proliferation. When the Wnt‐signal is on, the cytoplasmic β‐Catenin protein is stabilized and prevented from proteasomal degradation; β‐Catenin is translocated to the nucleus where it binds to LEF/TCF transcription factor and upregulates the expression of Wnt target genes involved in promoting the cell proliferation [44]. The expression of β‐Catenin was downregulated by KH‐3 in a time‐ and dose‐dependent manner. Further studies are needed to determine whether the antiproliferation activity of KH‐3 is fulfilled via inhibiting β‐Catenin.

KH‐3 causes apoptosis via a series of caspase activation events. BCL2 is an antiapoptotic protein; it maintains the integrity of the mitochondrial membrane by binding to and inhibiting the function of pro‐apoptotic proteins BAK/BAX, thus preventing the inside cytochrome c from releasing [45]. Otherwise, the released cytochrome c forms the apoptosome with Caspase‐9 and Apaf‐1 [46]. Therefore, anticancer reagents targeting BCL2‐regulated apoptosis expand treatment options for cancers. BH‐3 mimics are a class of anticancer reagents by neutralizing BCL2 homolog and releasing its inhibition on BAX/BAK [47]. Instead of arresting the function of BCL2, KH‐3 downregulates the protein level of BCL2 in a time‐ and dose‐dependent manner. Caspase inhibitors only partially rescued cells from KH‐3‐induced cell death, suggesting other cell death mode(s) that is/are independent of caspase activity. Our findings demonstrate a mechanism of action of KH‐3 possibly whereby inhibiting BCL2 as an early event to trigger apoptosis.

The combination of KH‐3 and docetaxel showed synergistic effects on reducing cell proliferation and tumor growth. The docetaxel single‐agent treatment did not alter the protein level of β‐Catenin and BCL2. However, KH‐3 treatment reduced their expression at the early stage of the treatment. Therefore, KH‐3 enhances the efficacy of docetaxel by inhibiting cell proliferation via attenuating the expression of β‐Catenin. On the other hand, KH‐3 restored the antitumor activity of docetaxel via inducing apoptosis triggered by BCL2‐regulated caspases activation in docetaxel‐resistant cells (Fig. 5B). The roles of BCL2 [48] and β‐Catenin [28, 49] involved in chemoresistance have been identified a long time ago by different research groups. Cell cycle analysis indicates that KH‐3 causes S‐phase arrest, preventing cells from entering mitosis. It has been reported that docetaxel caused cell death via inducing mitotic catastrophe [50]. It is possible that KH‐3 and docetaxel target different subpopulations of cells to induce synergistic effects, which needs to be determined by further studies. These results indicate a critical mechanism for docetaxel resistance in TNBC cells associated with increased cytoplasmic HuR accumulation and HuR‐regulated expression of BCL2, and β‐Catenin (Fig. 5B).

Triple‐negative breast cancer cells may employ multiple mechanisms simultaneously to acquire chemoresistance [28]. The ATP‐binding cassette (ABC) transporters are one of the important mechanisms. The multidrug‐resistant protein‐1 (ABCC1/MRP1) confers taxane resistance, and other ABC transporters share overlapping substrate specificity with it [28]. The mRNA levels of *ABCC1*, *ABCC4*, and *ABCC6* were increased at least twofold (Fig. S4B, and data not shown) in 231‐TR cells compared with MDA‐MB‐231 cells. KH‐3 treatment did not downregulate the mRNA levels of *ABCC1*, or *ABCC6*, except for *ABCC4* (Fig. 3D, and data not shown). And *ABCC4* was subsequently identified as a target of HuR, based on the RIP‐sequencing [17]. The possible schematic of how KH‐3 overcomes MRP4‐mediated chemoresistance was shown in Fig. 5B. Further investigation will determine whether KH‐3 interferes with drug transporter activity.

## 5 Conclusions

The results of this study highlight the crucial role of HuR in promoting docetaxel resistance, specifically, through its post‐transcriptional upregulating BCL2 and β‐Catenin, thereby providing a rationale for enhancing chemotherapy efficacy via the combination of HuR inhibitors and chemotherapeutic agents.

## Conflict of interest

The authors declare no conflict of interest.

## Author contributions

XW and LX contributed to the conceptualization, supervision, funding acquisition, review, revision, and approval of the manuscript. LW contributed to the investigation, methodology, validation, original draft preparation, editing, and revision of the manuscript. QZ, CZ, LH, YZ, AMA, JA, and DRW contributed to the investigation, methodology, review, and editing of the final manuscript. All authors have read and approved the final version of this manuscript.

## Supporting information


**Fig. S1.** Representative colony images of cells treated by KH‐3 or docetaxel.
**Fig. S2.** Fixed‐ratio combination MTT.
**Fig. S3.** Representative colony images of cells treated by KH‐3 and docetaxel.
**Fig. S4.** KH‐3 downregulated BCL2, β‐Catenin, and ABCC4.
**Fig. S5.** Cell cycle analysis of cells treated by KH‐3.
**Fig. S6.** Additional results for *in vivo* studies.
**Table S1.** Primers used for the RT‐qPCR.
**Table S2.** Primary antibody.
**Table S3.** Secondary antibody.
**Table S4.** Reagents.

## Data Availability

The data generated in this study are available within the paper and Supplementary information. Derived data supporting the findings of this study are available upon reasonable requests from the corresponding author.
