# Supplementary material for: Functional inhibition of the RNA‐binding protein HuR sensitizes triple‐negative breast cancer to chemotherapy
Source: Mol Oncol. 2023 Jul 19;17(10):1962–80. doi: 10.1002/1878-0261.13478 (PMC10552894; doi:10.1002/1878-0261.13478)
Supplement: Supplementary file 1 — Fig. S1. Representative colony images of cells treated by KH‐3 or docetaxel. Fig. S2. Fixed‐ratio combination MTT. Fig. S3. Representative colony images of cells treated by KH‐3 and docetaxel. Fig. S4. KH‐3 downregulated BCL2, β‐Catenin, and ABCC4. Fig. S5. Cell cycle analysis of cells treated by KH‐3. Fig. S6. Additional results for in vivo studies. Table S1. Primers used for the RT‐qPCR. Table S2. Primary antibody. Table S3. Secondary antibody. Table S4. Reagents. [file MOL2-17-1962-s001.docx]

# **Supplementary figures**


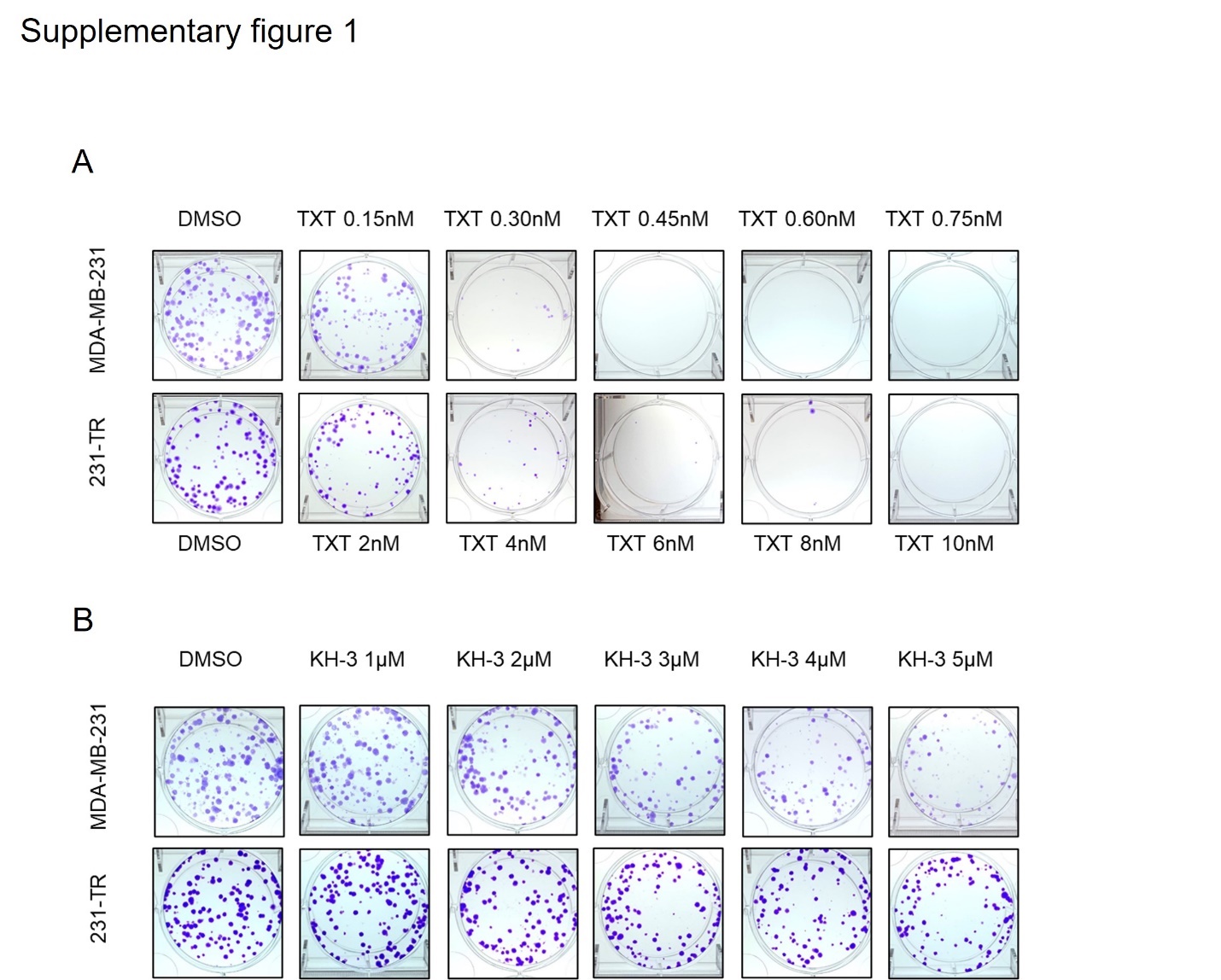


**Supplementary figure 1**. **Representative colony images of cells treated by KH-3 or docetaxel**. Representative images of colonies of MDA-MB-231 cells and 231-TR cells treated by docetaxel (**A**) or KH-3 (**B**). The experiment was performed in three repeats, and similar results were found.


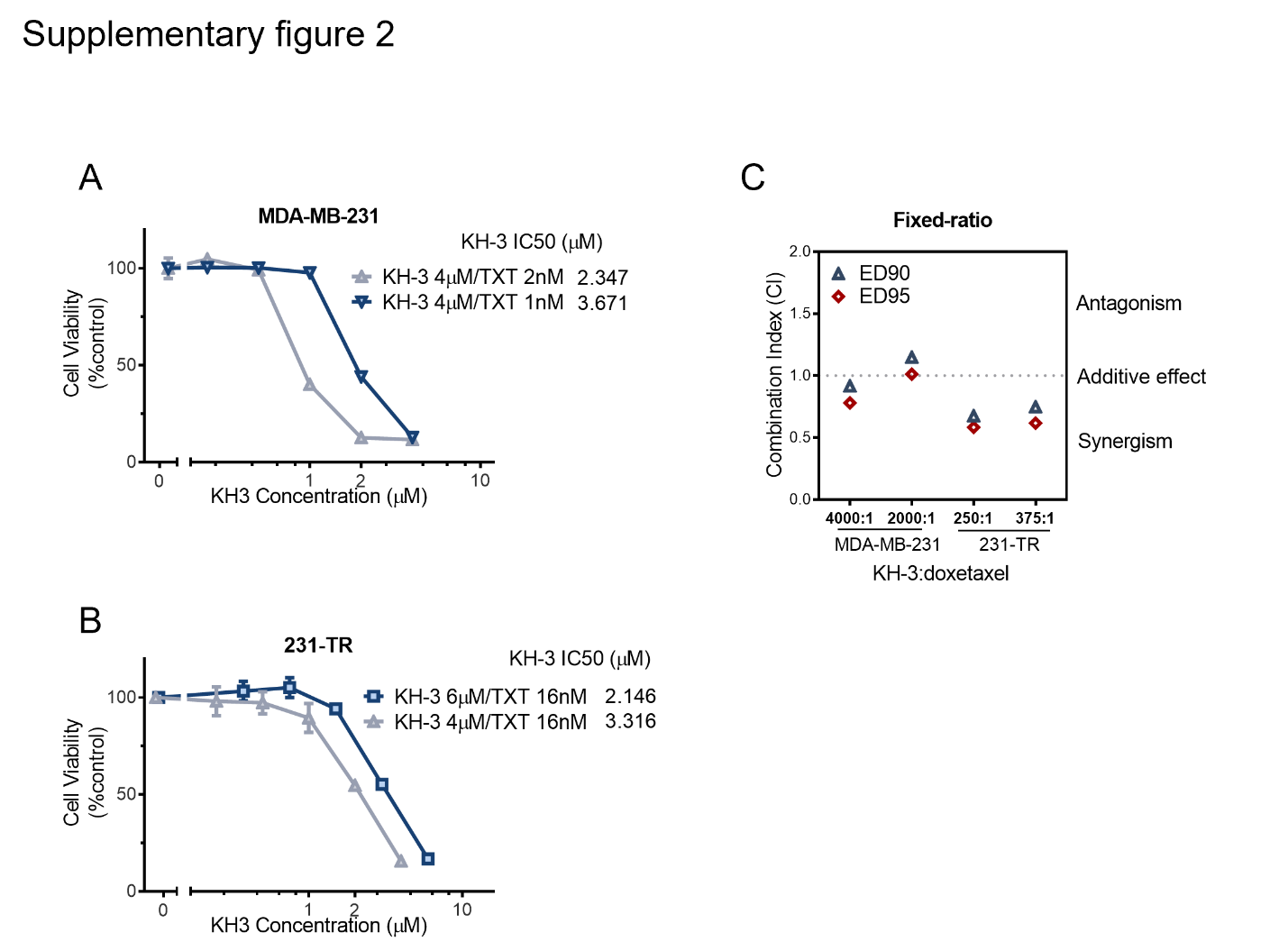


**Supplementary figure 2**. **Fixed-ratio combination MTT**. The combination-MTT-based cytotoxicity assay using a fixed ratio of KH-3 to docetaxel in (**A**) MDA-MB-231 cells and (**B**) 231-TR cells. Results are shown as mean ± SD of three replicates. (**C**) The combination index at different efficacy levels (ED90, and ED95) for the combination of KH-3 and docetaxel.


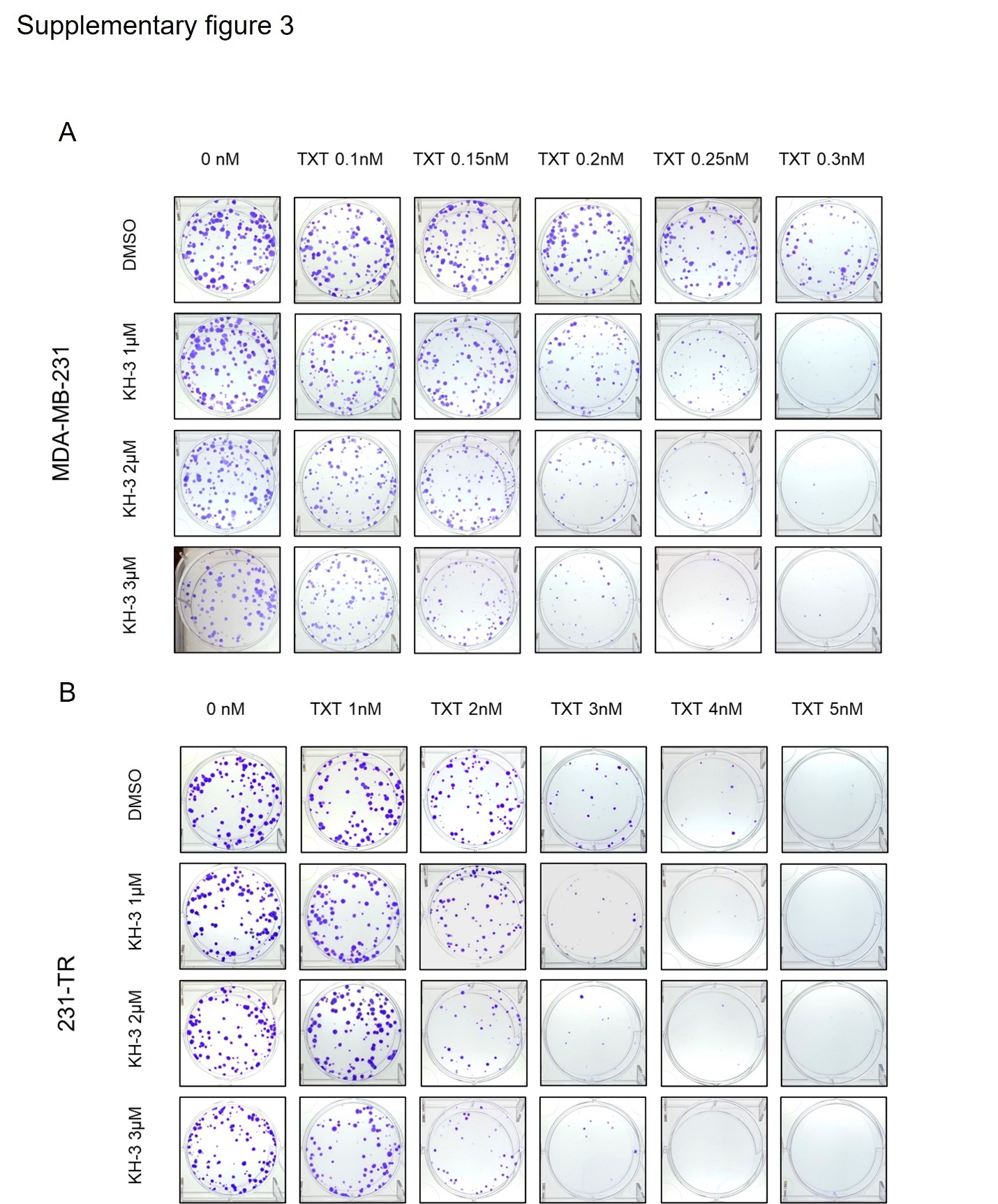


**Supplementary figure 3. Representative colony images of cells treated by KH-3 and docetaxel**. Representative images of colonies of cells receiving the combination treatment KH-3 plus docetaxel in (**A**) MDA-MB-231 cells and (**B**) 231-TR cells. The experiment was performed for three repeats, and similar results were found.


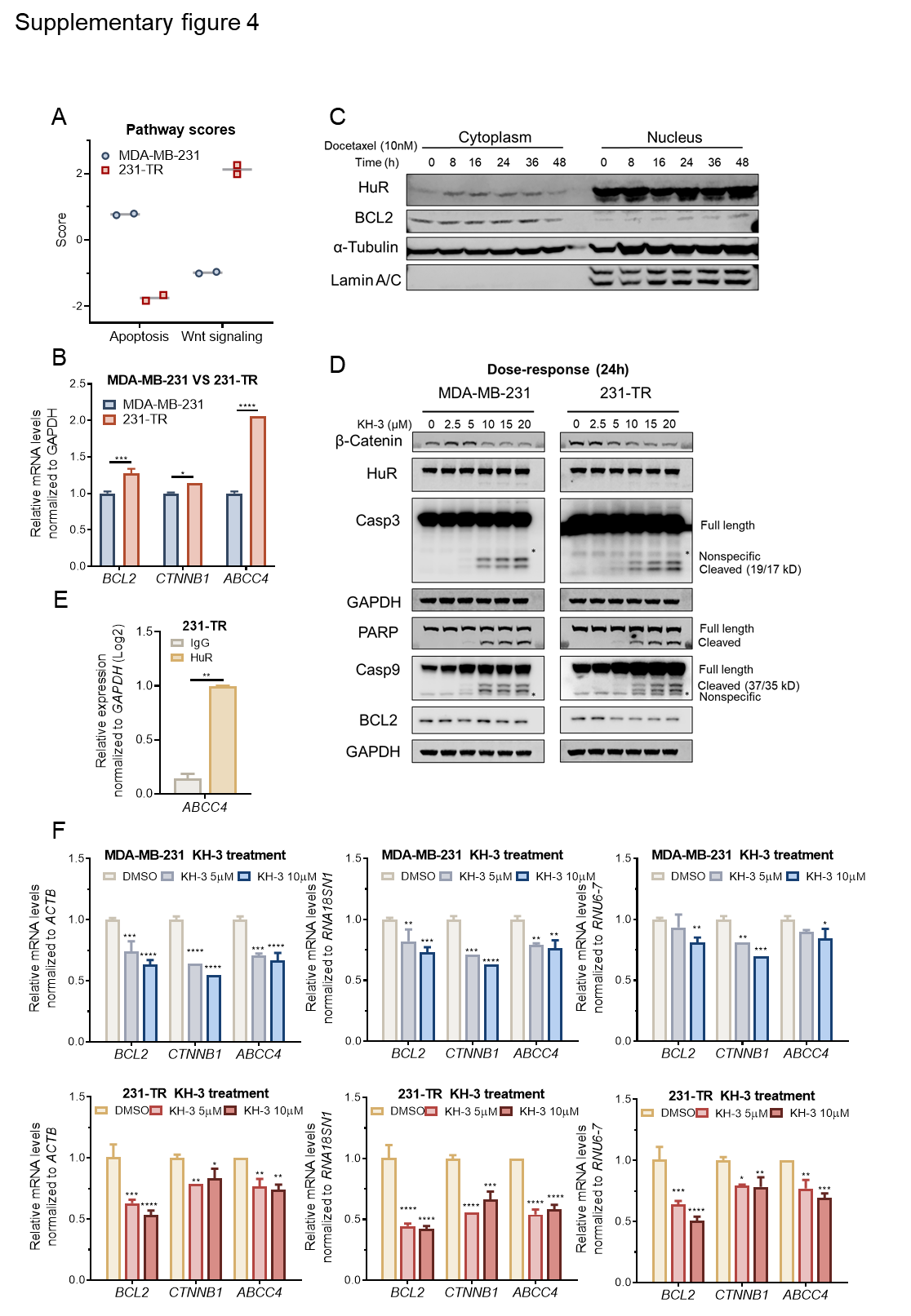


**Supplementary figure 4.** **KH-3 downregulated BCL2, β-Catenin, ABCC4**. (**A**) The scores of apoptosis, and Wnt signaling pathways of MDA-MB-231 cells and 231-TR cells, were shown as the mean and the individual value of two replicates. (**B**) RT-qPCR analysis of *BCL2*, *CTNNB1*, and *ABCC4* in 231-TR cells compared with MDA-MB-231 cells. Two-way ANOVA test, * *P* < 0.05, *** *P* < 0.001, **** *P* < 0.0001. Results are shown as mean ± SD of two replicates. (**C**) Western blot analysis of whole-cell lysates or cytoplasmic lysates from MDA-MB-231 cells treated by docetaxel for the indicated times. The α-Tubulin was used as the loading control. (**D**) Western blot analysis of whole-cell lysates from MDA-MB-231 cells or 231-TR cells treated with KH-3 from low to high concentration for 24h. GAPDH was used as the loading control. (**E**) RNP-IP analysis of HuR bound mRNAs in 231-TR cells. Cell lysates of 231-TR were subjected to RNP-IP followed by RT-qPCR analysis to measure the abundance of *ABCC4*. The enrichment of *ABCC4* in control IgG and HuR IP was compared. Unpaired t test, ** *P* < 0.01 (n = 2). (**F**) The RT-qPCR analysis on the mRNA levels of *BCL2*, *CTNNB1*, and *ABCC4* in MDA-MB-231 cells and 231-TR cells treated by KH-3 for 6h. Two-way ANOVA test, * P < 0.05, ** P< 0.01, *** P < 0.001, **** P < 0.0001 (n = 2). All results are shown as mean ± SD. All results were performed for three repeats, and the representative results were shown.


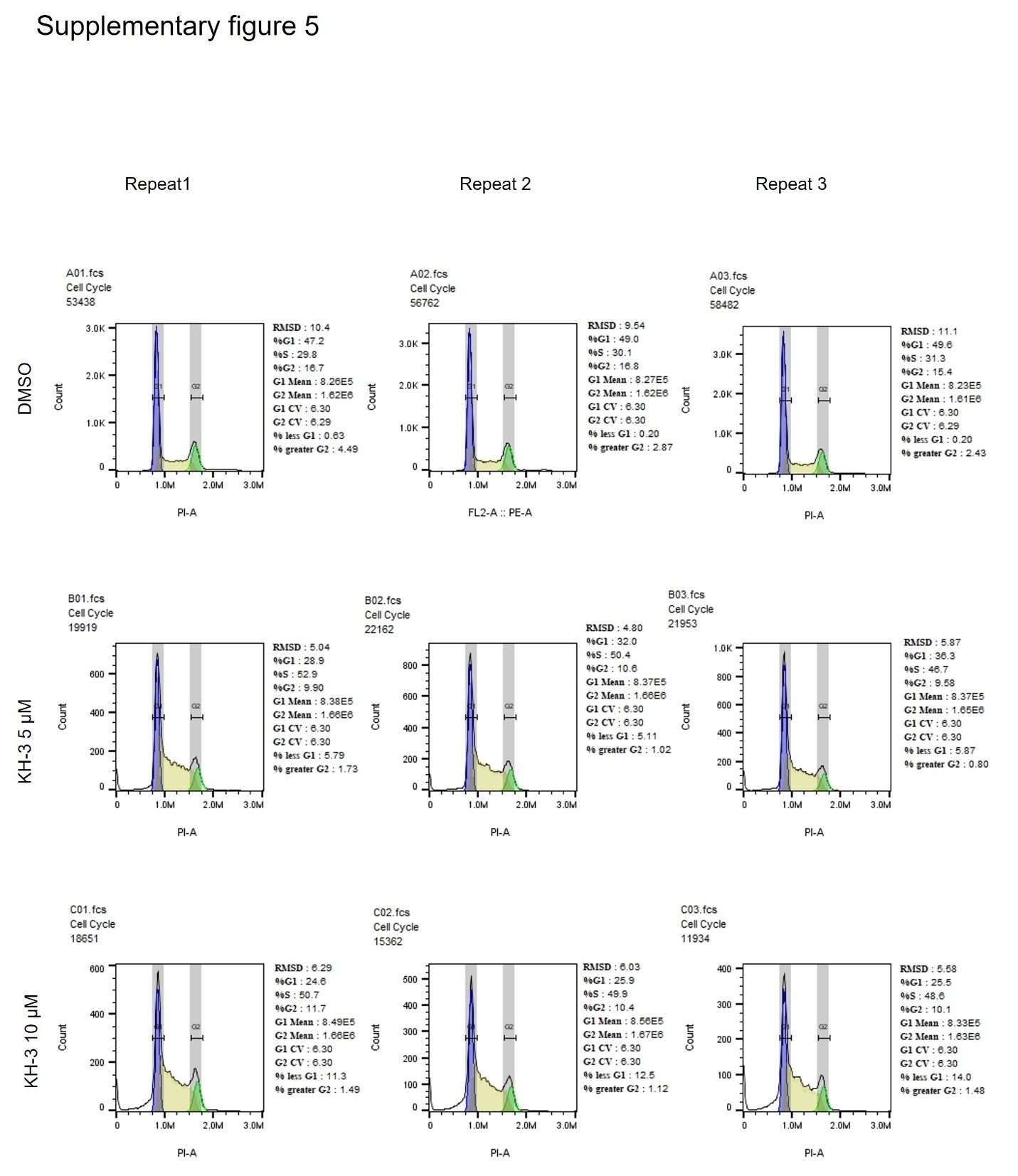


**Supplementary figure 5. Cell cycle analysis of cells treated by KH-3**. The histograms of cell cycle analysis and the percent of cell cycle analysis in MDA-MB-231 cells. The final event was indicated in the top left corner of each plot. The experiment was performed for three repeats, and the results were shown.


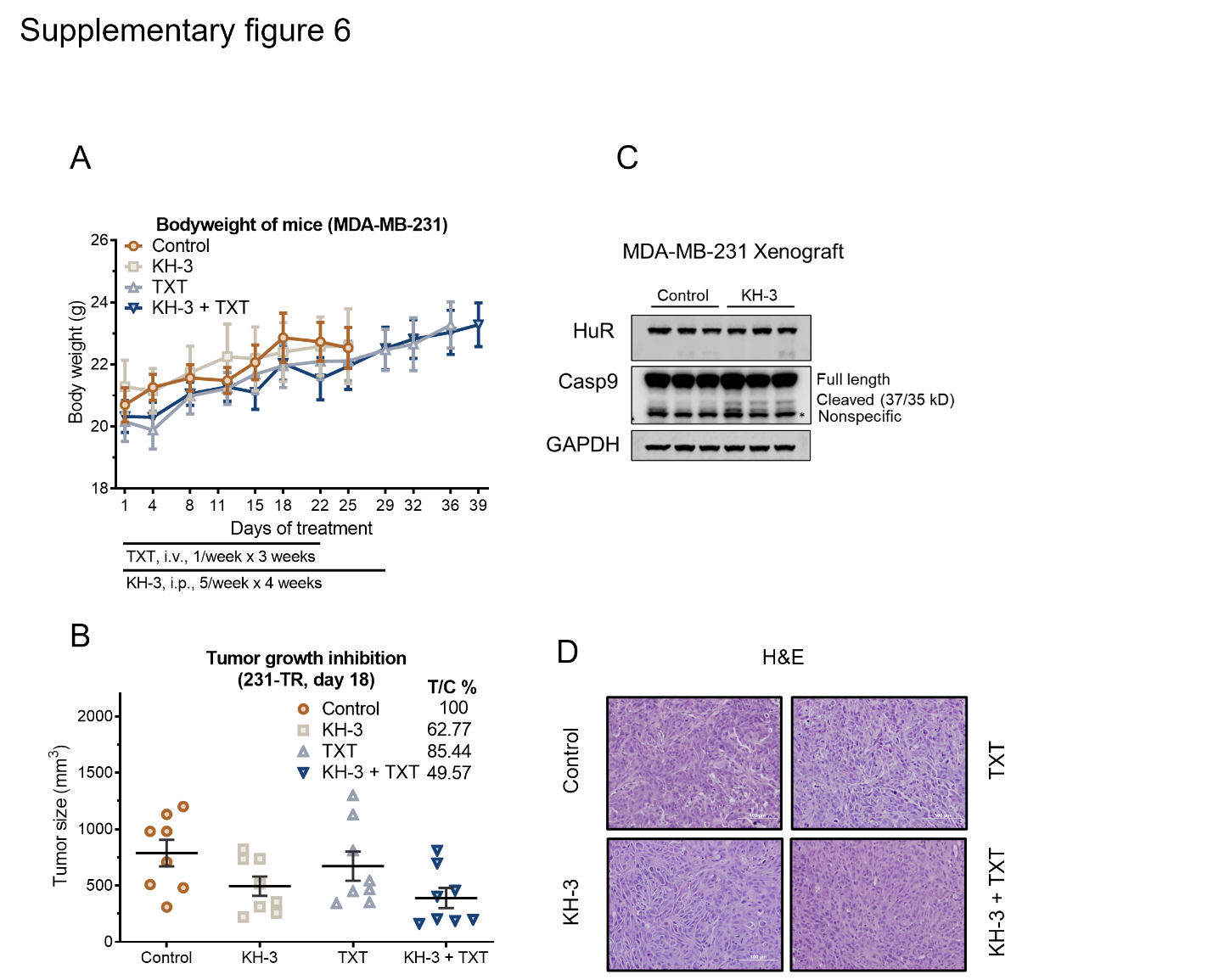


**Supplementary figure 6. Additional results for in vivo studies**. (**A**) Average body weight of mice bearing MDA-MB-231 xenografts and receiving the single agent or the combination treatment. Results are shown as mean ± SEM, n = 10. (**B**) The size of individual tumors in the 231-TR xenograft model at 18 days post-treatment. Results are shown as mean ± SEM. One-way ANOVA test, n = 8. (**C**) Western blot analysis of tissue lysates of (A) MDA-MB-231 xenografts. GAPDH was used as the loading control. (**D**) Representative images of immunohistology analysis on tumor tissues (Scale bar: 100 μM, Magnification: 400X). 231-TR xenografts were collected at the end of the treatment, fixed immediately using 4% formaldehyde, and then processed for H&E staining.

# **Supplementary tables**

| **Supplementary table 1. Primers used for the RT-qPCR** | | |
| --- | --- | --- |
| **Gene** | **Sequences** |  |
| *ABCC4* | Forward: 5'- ACGCGAACCTCTGCTCAC -3' |  |
|  | Reverse: 5'- CTCCTCTCCAAGGTGCTGTG -3' |  |
| *BCL2* | Forward: 5'- CCCTGTGGATGACTGAGTACC -3' |  |
|  | Reverse: 5'- GCCGTACAGTTCCACAAAGG -3' |  |
| *CTNNB1* | Forward: 5'- ATGGCTTGGAATGAGACTGCT -3' |  |
|  | Reverse: 5'- GGGTCCATACCCAAGGCATC -3' |  |
| *GAPDH* | Forward: 5'- ATGTTCGTCATGGGTGTGAA -3' |  |
|  | Reverse: 5'- GGTGCTAAGCAGTTGGTGGT -3' |  |
| *ACTB* | Forward: 5'- CTCTTCCAGCCTTCCTTCCT -3' |  |
|  | Reverse: 5'- AGCACTGTGTTGGCGTACAG -3' |  |
| *RNA18SN1* | Forward: 5'- GTAACCCGTTGAACCCCATT -3' |  |
|  | Reverse: 5'- CCATCCAATCGGTAGTAGCG -3' |  |
| *RNU6-7* | Forward: 5'- CTCGCTTCGGCAGCACA -3' |  |
|  | Reverse: 5'- AACGCTTCACGAATTTGCGT -3' |  |

| **Supplementary table 2. Primary antibody** | | |
| --- | --- | --- |
| **Target** | **Cat#** | **Source** |
| β-Catenin | sc-7963 | Santa Cruz |
| BCL2 | 4223s | Cell Signaling Technology |
| Caspase 3 | 9662s | Cell Signaling Technology |
| Caspase 9 | 9502s | Cell Signaling Technology |
| PARP | 9542s | Cell Signaling Technology |
| HuR | sc-5261 | Santa Cruz |
| Lamin A/C | 4777s | Cell Signaling Technology |
| GAPDH | 5174s | Cell Signaling Technology |
| α-tubulin | T5168 | Sigma |

| **Supplementary table 3. Secondary antibody** | | |
| --- | --- | --- |
| **Target** | **Cat#** | **Source** |
| 680RD Goat anti-Rabbit | 926-68071 | LI-COR Biosciences |
| 680RD Goat anti-Mouse | 926-68070 | LI-COR Biosciences |
| 800CW Goat anti-Rabbit | 926-32211 | LI-COR Biosciences |
| 800CW Goat anti-Mouse | 926-32210 | LI-COR Biosciences |

| **Supplementary table 4. Reagents** | | |
| --- | --- | --- |
| **reagent** | **Cat#** | **Source** |
| Z-VAD-FMK | S7023 | Selleck Chem |
| Q-VD-Oph | S7311 | Selleck Chem |
| Methanol | A452-4 | Fisher Chemical |
| Chloroform | C607-1 | Fisher Scientific |
| Ethanol | 64-17-5 | Decon Laboratories |
| 2-Propanol | 190764-1L | Sigma-Aldrich |
| Dimethyl sulfoxide | 472301 | Sigma-Aldrich |
| Phosphate Buffered Saline | P4417-100TAB | Sigma |
| Propidium iodide | P4170 | Sigma-Aldrich |
| RNAse | 1007885 | Qiagen |
